# Assessing the relationship between quality of life and behavioral activation using the Japanese Behavioral Activation for Depression Scale-Short Form

**DOI:** 10.1371/journal.pone.0185221

**Published:** 2017-09-28

**Authors:** Yusuke Shudo, Tatsuya Yamamoto

**Affiliations:** 1 Faculty of Psychology, Hiroshima International University, Hiroshima, Japan; 2 School of Psychology, Chukyo University, Aichi, Japan; Chiba Daigaku, JAPAN

## Abstract

Quality of life (QOL) is an important health-related concept. Identifying factors that affect QOL can help develop and improve health-promotion interventions. Previous studies suggest that behavioral activation fosters subjective QOL, including well-being. However, the mechanism by which behavioral activation improves QOL is not clear. Considering that QOL improves when depressive symptoms improve post-treatment and that behavioral activation is an effective treatment for depression, it is possible that behavioral activation affects QOL indirectly rather than directly. To clarify the mechanism of the influence of behavioral activation on QOL, it is necessary to examine the relationships between factors related to behavioral activation, depressive symptoms, and QOL. Therefore, we attempted to examine the relationship between these factors. Participants comprised 221 Japanese undergraduate students who completed questionnaires on behavioral activation, QOL, and depressive symptoms: the Japanese versions of the Behavioral Activation for Depression Scale-Short Form (BADS-SF), WHO Quality of Life-BREF (WHOQOL-26), and Center for Epidemiologic Studies Depression Scale (CES-D). The BADS-SF comprises two subscales, Activation and Avoidance, and the WHOQOL-26 measures overall QOL and four domains, Physical Health, Psychological Health, Social Relationships, and Environment. Mediation analyses were conducted with BADS-SF activation and avoidance as independent variables, CES-D as a mediator variable, and each WHO-QOL as an outcome variable. Results indicated that depression completely mediated the relationship between Avoidance and QOL, and partially mediated the relationship between Activation and QOL. In addition, analyses of each domain of QOL showed that Activation positively affected all aspects of QOL directly and indirectly, but Avoidance had a negative influence on only part of QOL mainly through depression. The present study provides behavioral activation strategies aimed at QOL enhancement.

## Introduction

Quality of life (QOL) is defined as “individuals’ perception of their position in life in the context of the culture and value systems in which they live and in relation to their goals, expectations, standards and concerns,” (see [1]), and includes concepts of subjective well-being and happiness. Currently, QOL has been actively researched in the field of mental health, particularly its relationship to major depressive disorders [2], anxiety disorders [3], and schizophrenia [4].

As depression is still a serious concern, effective and inexpensive treatments continue to be explored. In particular, behavioral activation treatment for depression has attracted attention. According to behavioral theory underlying behavior activation treatment, avoidance behavior plays an important role in the occurrence and maintenance of depression [5]. In a stressful environment, avoidance of unpleasant events increases, such as postponement and social withdrawal, and behaviors other than avoidance decrease accordingly. As a result, adaptive behaviors related to a healthy lifestyle are decreased, and depression develops and is maintained. Behavioral activation for depression attempts to increase adaptive behavior and decrease avoidance behaviors [6] by using simple activation methods such as short-term goals and activity schedules, and avoidance interventions such as monitoring of avoidance behavior and engaging in alternative behaviors instead of avoidance. The Behavioral Activation for Depression Scale [7] and its short version (BADS-SF) [8] were developed to assess changes in behavior resulting from behavioral activation. The BADS-SF comprises two subscales, Activation (trait assumed to vary by simple activation technique) and Avoidance (trait assumed to vary by interventions for avoidance behavior).

Several studies confirm the effectiveness of behavioral activation as a psychological therapy for depression [9–11]. However, as aforementioned, one of the purposes of behavioral activation is to increase the frequency of adaptive behavior in order to increase the likelihood of receiving positive reinforcement [5]. Such procedures are considered beneficial not only for depressed people but also for healthy individuals. Accordingly, Mazzucchelli et al.’s [12] meta-analysis revealed that behavioral activation promotes subjective well-being. Additionally, the benefits of a group intervention involving behavioral activation and mindfulness therapy for subjective well-being were confirmed [13]. Collectively, these studies suggest that behavioral activation promotes subjective well-being. However, the relationship between factors related to behavioral activation and QOL, subjective well-being, and happiness has received little attention. Identifying factors that affect QOL can help develop and improve health-promotion interventions.

Considering that QOL improves when depressive symptoms improve post-treatment [14] and that behavioral activation is an effective treatment for depression, it is possible that behavioral activation does not directly affect QOL. On the other hand, in the study of behavioral activation and mindfulness for non-clinical populations, improvement of well-being has been shown [13], suggesting that behavioral activation may directly affect QOL. Therefore, there is a possibility that variables related to behavioral activation directly affect QOL or that they indirectly affect it by mediating depression. Thus, the purpose of this study was to examine whether depression mediates the influence of behavioral activation on QOL, through mediation analysis.

## Materials and methods

### Ethics statement

The Research Ethics Committee of Chukyo University approved all methods and study procedures, which were in accordance with the Declaration of Helsinki and its later amendments. Prior to data collection, participants were given a complete description of the study, including the risks and benefits of participation, after which, written informed consent was sought.

### Participants and procedure

A total of 221 Japanese undergraduate students (93 women and 128 men; age: *M* = 19.37, *SD* = 1.74, range = 18–25) participated in a questionnaire study. A demographic questionnaire on age and gender (*male/female*), and the Japanese BADS-SF, Japanese CES-D, and WHOQOL-26 were administered to participants during their classes (primarily during psychology lectures). Participants were not compensated.

### Measures

#### BADS-SF

The BADS-SF [8] is a 9-item shortened version of the original scale [7] designed to assess changes in activation and avoidance behavior, based on the behavioral activation theory. All items (e.g., “There were certain things I needed to do that I didn’t do”) are rated on a 7-point Likert scale ranging from 0 “*Not at all*” to 6 “*Completely*.” The Activation subscale comprises six items, while Avoidance contains three. Yamamoto et al.’s [15] Japanese translation, which demonstrates good reliability (Activation: Cronbach’s α = .79; Avoidance: α = .71; Total: α = .71) and validity, was employed. The Japanese BADS-SF scores (Activation: 5 items; Avoidance: 3 items) are computed by summing subscale scores after reverse-scoring three negatively worded items. High total scores indicate self-reported high activity levels and low avoidant-behavior levels. Activity and Avoidance subscales are scored such that high scores indicate high activity levels and high avoidance behavior, respectively.

#### CES-D

The CES-D [16] is a 20-item self-report scale that measures depressive symptomatology. Each item (e.g., “I was bothered by things that usually don’t bother me”) is rated on a 4-point rating scale, ranging from 0 “*Rarely or never*” to 3 “*Most of the time or always*,” to reflect how the individual felt during the week prior to testing. Shima et al. [17] translated the CES-D into Japanese, and confirmed its reliability (test-retest reliability: *r* = .84, Spearman-Brown’s split-half reliability: *r* = .79) and validity. The total depressive-symptoms score is computed by reverse-scoring four negatively worded items and summing all items, with a theoretical range of 0–60.

#### WHOQOL-26

The WHOQOL-26 is the Japanese version of the WHO Quality of Life-BREF [18], translated and tested for equivalence by Tazaki and Nakane [19]. The WHOQOL-26 comprehensively measures subjective well-being; it comprises 24 items in four domains—Physical Health, Psychological Health, Social Relationships, and Environment—and two items for Overall QOL and General Health. Each item (e.g., “To what extent do you feel that physical pain prevents you from doing what you need to do?”) is rated on a 5-point Likert scale ranging from 1 (e.g., *Not at all/Very dissatisfied*) to 5 (e.g., *A lot/Very satisfied*). It demonstrates adequate reliability (Cronbach’s α = .66–.84) and discriminant validity.

### Statistical analyses

Gender differences in BADS-SF total and subscale scores, the CES-D score, and mean total and domain WHOQOL-26 scores were examined with *t*-tests. Zero-order correlations were performed to examine the relationships between measures.

Several studies [7,8] have revealed that variables related to behavioral activation affect depression, and it is also well known that depression affects QOL [14]. Therefore, variables related to behavioral activation may affect QOL through mediation of depression. To clarify the mechanism of QOL and behavioral activation, it is necessary to clarify the relationship between variables related to behavioral activation, depression, and QOL. In this study, mediation analyses were conducted using a bootstrapping method. The bootstrapping method was performed in line with recommendations by Preacher and Hayes [20], with k = 5000 resamples and 95% bias-corrected and accelerated (BCa) confidence intervals (CI) used to evaluate indirect effects. An indirect effect is determined to be statistically significant if the CI does not contain zero.

In each analysis, BADS-SF Activation and Avoidance scores were the independent variable (X), CES-D score was the mediator variable (M), and each WHO-QOL score was the outcome variable (Y). In the first set of analyses, the WHOQOL-26 Mean Total Score was Y. Similar analysis was performed on a model with the domain- and overall-QOL scores as Y. The conditions for judging that a mediating effect has occurred are as follows [20, 21]; (1) influence of X on Y (total effect) is significant; (2) indirect effect is significant; (3) the absolute value of the direct effect decreases compared to the total effect. In the complete mediation, the direct effect of X on Y is not significant, but the indirect effect is significant. On the other hand, in partial mediation, both direct and indirect effects of X on Y are significant. All analyses were conducted using R version 3.4.1 and lavaan package version 0.5–23.

## Results

Descriptive statistics and zero-order correlations for each scale are presented in Tables 1 and 2. There were no significant gender differences except in the Activation subscale. Therefore, analyses were conducted without considering gender differences.

**Table 1:** Descriptive statistics of participants’ scores by gender.

	Total Sample (*N* = 221)					Men (*n* = 128)		Women (*n* = 93)		*t*
	*M*	*SD*	*α*	Skew.	Kurt.	*M*	*SD*	*M*	*SD*	
**1. CES-D**	15.41	8.05	0.81	0.92	1.40	14.80	7.91	16.24	8.20	-1.30
**2. BADS-SF Total**	23.51	8.09	0.78	-0.04	0.24	24.01	8.90	22.82	6.79	1.12
**3. Activation**	13.02	5.93	0.83	0.06	-0.15	13.75	6.34	12.01	5.18	2.24*
**4. Avoidance**	7.51	4.02	0.68	0.57	-0.02	7.74	4.44	7.19	3.34	1.05
**5. WHOQOL-26** **Mean Total Score**	3.31	0.56	0.91	0.05	0.47	3.33	0.60	3.28	0.50	0.71
**6. Physical Health**	3.45	0.62	0.69	-0.07	-0.09	3.51	0.63	3.35	0.59	1.92
**7. Psychological Health**	3.15	0.74	0.79	-0.18	0.22	3.21	0.79	3.07	0.66	1.52
**8. Social Relationships**	3.32	0.77	0.72	-0.15	0.16	3.25	0.86	3.42	0.62	-1.70
**9. Environment**	3.31	0.63	0.77	-0.25	1.53	3.30	0.66	3.33	0.58	-0.38
**10. Overall QOL**	3.23	0.83	0.58	-0.18	0.13	3.27	0.90	3.19	0.72	0.66

CES-D, Center for Epidemiologic Studies Depression Scale; BADS-SF, Behavioral Activation for Depression Scale-Short Form; WHOQOL-26, Japanese version of the WHO Quality of Life-BREF; QOL, Quality of Life; Skew., Skewness; Kurt., Kurtosis; α, Cronbach’s α.

*p < .05.

**Table 2:** Correlations between measures (*N* = 221).

	1	2	3	4	5	6	7	8	9
**1. CES-D**									
**2. BADS-SF Total**	-0.55**								
**3. Activation**	-0.40**	0.88**							
**4. Avoidance**	0.53**	-0.71**	-0.30**						
**5. WHOQOL-26** **Mean Total Score**	-0.58**	0.59**	0.54**	-0.39**					
**6. Physical Health**	-0.49**	0.51**	0.44**	-0.38**	0.86**				
**7. Psychological Health**	-0.64**	0.65**	0.58**	-0.45**	0.86**	0.68**			
**8. Social Relationships**	-0.42**	0.39**	0.38**	-0.22**	0.74**	0.54**	0.60**		
**9. Environment**	-0.37**	0.36**	0.36**	-0.20**	0.84**	0.60**	0.57**	0.55**	
**10. Overall QOL**	-0.45**	0.52**	0.50**	-0.31**	0.71**	0.56**	0.59**	0.47**	0.51**

CES-D, Center for Epidemiologic Studies Depression Scale; BADS-SF, Behavioral Activation for Depression Scale—Short Form WHOQOL-26, Japanese version of the WHO Quality of Life-BREF; QOL, Quality of Life.

*p < .05.

**p < .01.

**Fig 1:**
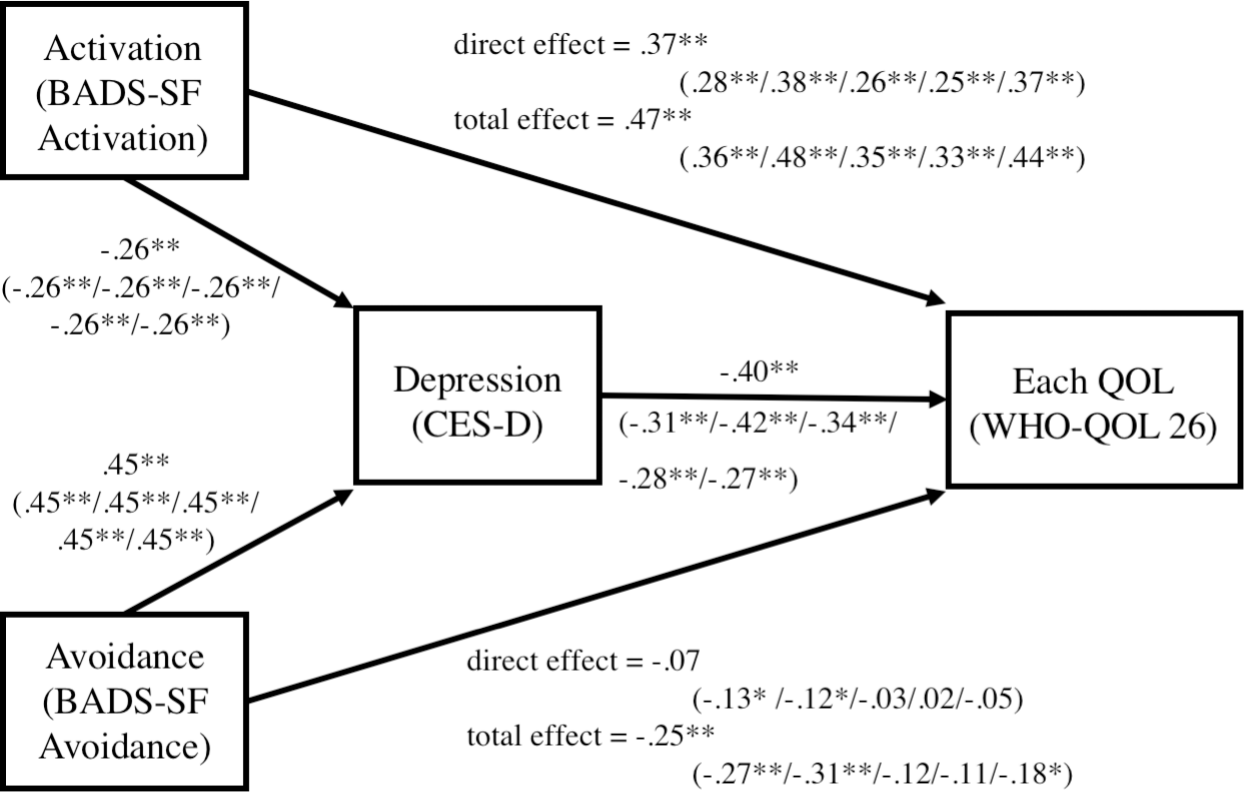
Mediating effect of depression on the relationship between behavioral-activation related factors and QOL. Note. Each value indicates standardized path coefficients when each QOL was used as a target variable. WHOQOL-26 Mean Total Score (Physical Health/Psychological Health/Social Relationships/Environment/Overall QOL). BADS-SF: Behavioral Activation for Depression Scale Short form; CES-D: Center for Epidemiologic Studies Depression Scale; QOL: Quality of Life. **p < .01, *p < .05.

In order to examine the hypothesis that depression mediates the relationship between a behavioral activation-related factor and QOL, mediation analysis was conducted (see Fig 1). In the first set of analyses, the WHOQOL-26 Mean Total Score was Y. The effects of Activation and Avoidance on the WHOQOL-26 Mean Total Score were significant (total effect: Activation *B* = .05, *β* = .47, *SE* = .00, *p* < .01; Avoidance *B* = -.04, *β* = .25, *SE* = .00, *p* < .01). When controlling CES-D scores as mediating variables, the effect of Activation on the WHOQOL-26 Mean Total Score was significant (direct effect: *B* = .04, *β* = .37, *SE* = .01, *p* < .01), but Avoidance was not (direct effect: *B* = -.01, *β* = -.07, *SE* = .01, *p* = .26), and the coefficient of each direct effect was decreased compared to that of the total effect. In terms of the effect of X on M, the standardized regression coefficients from Activation and Avoidance to CES-D were -.26 (*p* < .01) and .45 (*p* < .01). In terms of the effect of M on Y, the coefficient from CES-D to the WHOQOL-26 Mean Total Score was .40 (*p* < .01). Indirect effects were calculated with the bootstrapping method. The indirect effects of Activation and Avoidance on the WHOQOL-26 Mean Total Score were significant (indirect effect: Activation *B* = .01, *β* = .10, 95% CI = .01 to .02; Avoidance *B* = -.03, *β* = -.18, 95% CI = -.04 to -.02). Therefore, this result indicated that depression partially mediates the relationship between Activation and QOL, and completely mediates the relationship between Avoidance and QOL.

Subsequently, similar analyses were performed with the domain- and overall-QOL scores as the outcome variable Y. In the domain of QOL, Physical Health and Psychological Health showed different results from Social Relationship and the environment. The effect of Activation and Avoidance on Physical Health (total effect: Activation *B* = .04, *β* = .36, *SE* = .01, *p* < .01; Avoidance *B* = -.04, *β* = -.27, *SE* = .01, *p* < .01) and Psychological Health (total effect: Activation *B* = .01, *β* = .11, *SE* = .01, *p* < .01; Avoidance *B* = -.06, *β* = -.31, *SE* = .01, *p* < .01) were significant. When controlling CES-D score as mediating variables, the effect of Activation and Avoidance on Physical Health (direct effect: Activation *B* = .03, *β* = .28, *SE* = .01, p < .01; Avoidance *B* = -.02, *β* = -.13, *SE* = .01, *p* < .05) and Psychological Health (direct effect: Activation *B* = .05, *β* = .38, *SE* = .01, *p* < .01; Avoidance *B* = -.02, *β* = -.12, *SE* = .01, *p* < .05) were significant, and the coefficient of each direct effect was decreased compared to that of the total effect. The indirect effect of Activation and Avoidance on Physical Health (indirect effect: Activation *B* = .01, *β* = .08, 95% CI = .00 to .02; Avoidance *B* = -.02, *β* = -.14, 95% CI = -.03, -.01) and Psychological Health (indirect effect: Activation *B* = .01, *β* = .11, 95% CI = .01 to .02; Avoidance *B* = -.04, *β* = -.19, 95% CI = -.05 to -.03) were significant. Therefore, this result indicated that depression partially mediates relationship between factors related to behavioral activation and QOL, in the domain of Physical Health and Psychological Health.

On the other hand, the effect of Activation on Social Relationships (total effect: Activation *B* = .05, *β* = .35, *SE* = .01, *p* < .01) and Environment (total effect: Activation *B* = .04, *β* = .33, *SE* = .01, *p* < .01) were significant, but Avoidance on Social Relationships (total effect: Avoidance *B* = -.02, *β* = -.12, *SE* = .01, *p* = .08) and Environment (total effect: Avoidance B = -.02, *β* = -.11, *SE* = .01, *p* = .16) were not significant. Therefore, in Avoidance, condition (1) of judging that a mediating effect occurred was not satisfied. When controlling CES-D scores, the effect of Activation on Social Relationships (direct effect: Activation *B* = .03, *β* = .26, *SE* = .01, *p* < .01) and Environment (direct effect: Activation *B* = .03, *β* = .25, *SE* = .01, *p* < .01) were significant and the coefficient of each direct effect was decreased compared to that of the total effect. The indirect effect of Activation on Social Relationships (indirect effect: Activation *B* = .01, *β* = .09, 95%CI = .01 to .02) and Environment (indirect effect: Activation *B* = .01, *β* = .07, 95%CI = .00 to .01) were significant. This result indicates not only that depression does not mediate the effect of Avoidance on QOL, but also that Avoidance does not affect QOL. At the same time, Activation affects QOL directly and indirectly.

For Overall QOL, the effect of Activation and Avoidance were significant (total effect: Activation *B* = .06, *β* = .44, *SE* = .01, *p* < .01; Avoidance *B* =.-.04, *β* = -.18, *SE* = .01, *p* < .05). When controlling CES-D scores, the effect of Activation was significant, but Avoidance was not (direct effect: Activation *B* = .05, *β* = .37, SE = .01, *p* < .01; Avoidance *B* = -.01, *β* = -.05, SE = .02, *p* = .47), and the coefficient of each direct effect was decreased compared to that of the total effect. The indirect effect of Activation and Avoidance were significant (indirect effect: Activation *B* = .01, *β* = .07, 95% CI = .00 to .01; Avoidance *B* = -.03, *β* = -.12, 95% CI = -.04 to -.01). Similarly, in the case of the WHOQOL-26 Mean Total Score, depression partially mediates relationship between Activation and overall QOL, and completely mediates the relationship between Avoidance and overall QOL.

## Discussion

The present study examined whether depression mediates the influence of a behavioral activation factor on QOL. Mediation analysis showed that Activation indirectly affected QOL through depression and directly affected QOL without mediation of depression at the same time, but Avoidance affected QOL only through depression. In addition, the effect of Activation on depression was weaker than that of Avoidance, but the total effect on QOL was stronger. This result shows the importance of Avoidance in depression, as previous studies [5] have pointed out, and newly indicates that Activation plays an important role in QOL.

In addition, the analysis of each domain of QOL showed that Activation, directly and indirectly through depression, affected all domains, but the effect of Avoidance was complicated. For Physical health and Psychological health, Avoidance directly and indirectly affected QOL and showed partial mediation through depression. In contrast, the total effect of Avoidance on QOL for Environmental and Social relationships was not significant; these results suggest Avoidance did not affect QOL. On the other hand, for Overall QOL, which is a subjective general sense of QOL, Avoidance showed complete mediation and affected QOL through depression. These results show that Activation positively affects all aspects of QOL directly and indirectly, but that Avoidance has a negative influence only on a part of QOL mainly through depression.

Previous research shows that improvements in depressive symptoms enhance QOL [14]. If only alleviation of depression enhances QOL, improved QOL is merely an additional depression-treatment outcome, and it is considered that QOL improvement by behavioral activation only occurs for individuals with depression. Conversely, for healthy individuals who do not have depressive symptoms, it is not possible to bring further depression improvement, so it can be said that QOL improvement by behavioral activation does not occur. However, the results of the present study showed that avoidance was associated with QOL through depressive symptoms, but activation directly affected QOL. Taking this result into account, behavioral activation—particularly activation—is associated with QOL independent of depression, suggesting that simple activation enhances QOL in both healthy individuals and those with depression. This finding is consistent with Mazzucchelli et al.’s [13] study wherein behavioral activation and mindfulness therapy conducted with a community-based non-clinical adult sample was found to improve several indices of well-being.

The present findings can inform behavioral activation strategies aimed at QOL enhancement. Recently, avoidance behavior has become the focus of depression treatment, which is central to the contemporary behavioral activation treatment model [22]. There is some evidence to suggest that modern behavioral activation may be more effective for depression than its earlier variants [23]. On the other hand, it is possible that interventions for avoidance behavior aimed at QOL enhancement have only limited effects and are less effective than a simple activation intervention.

Previous studies have shown that behavioral activation is effective for improving both depression and well-being [12,13], and the results of this study also suggest similar conclusions. However, improved strategies—rather than conventional behavioral activation methods—could increase the efficacy of QOL enhancement. Considering that the present study found a positive association between activation and all aspects of QOL, behavioral activation for health promotion should emphasize simple activation.

The present study is limited in terms of the findings’ generalizability, as the sample comprised only university students. The results—suggesting that activation rather than avoidance is related to QOL—must be interpreted with caution, especially when attempting to extend them to clinical samples.

We hope that the present study can help inform health-promotion development initiatives by clarifying the mechanisms of QOL.

## References

[1] WHOQOL-Group. Study protocol for the World Health Organization project to develop a Quality of Life assessment instrument (WHOQOL). Qual Life Res. 1993;2:153–9. doi: 10.1007/BF00435734 8518769

[2] Kennedy SH, Eisfeld BS, Cooke RG. Quality of life: an important dimension in assessing the treatment of depression? J Psychiatry Neurosci. 2001;26 Suppl:S23–8. 11590966 PMC2553258

[3] Barrera TL, Norton PJ. Quality of life impairment in generalized anxiety disorder, social phobia, and panic disorder. J Anxiety Disord. 2009;23:1086–90. doi: 10.1016/j.janxdis.2009.07.011 19640675 10.1016/j.janxdis.2009.07.011 PMC2782397

[4] Galuppi A, Turola MC, Nanni MG, Mazzoni P, Grassi L. Schizophrenia and quality of life: how important are symptoms and functioning? Int J Ment Health Syst. 2010;4:31 doi: 10.1186/1752-4458-4-31 21143871 10.1186/1752-4458-4-31 PMC3016370

[5] Martell CR, Dimidjian S, Herman-Dunn R. Behavioral activation for depression: A clinician’s guide New York: The Guilford Press; 2010.

[6] Manos RC, Kanter JW, Busch AM. A critical review of assessment strategies to measure the behavioral activation model of depression. Clin Psychol Rev. 2010;30(5):547–61. doi: 10.1016/j.cpr.2010.03.008 20444531 10.1016/j.cpr.2010.03.008

[7] Kanter JW, Mulick PS, Busch AM, Berlin KS, Martell CR. The Behavioral Activation for Depression Scale (BADS): psychometric properties and factor structure. J Psychopathol Behav Assess. 2007;29:191–202. doi: 10.1007/s10862-006-9038-5

[8] Manos RC, Kanter JW, Luo W. The Behavioral Activation for Depression scale-Short Form: development and validation. Behav Ther. 2011;42(4):726–39. doi: 10.1016/j.beth.2011.04.004 22036000 10.1016/j.beth.2011.04.004

[9] Dimidjian S, Hollon SD, Dobson KS, Schmaling KB, Kohlenberg RJ, Addis ME, et al Randomized trial of behavioral activation, cognitive therapy, and antidepressant medication in the acute treatment of adults with major depression. J Consult Clin Psychol. 2006;74(4):658–70. doi: 10.1037/0022-006X.74.4.658 16881773 10.1037/0022-006X.74.4.658

[10] Cuijpers P, Andersson G, Donker T, van Straten A. Psychological treatment of depression: results of a series of meta-analyses. Nord J Psychiatry. 2011;65(6):354–64. doi: 10.3109/08039488.2011.596570 21770842 10.3109/08039488.2011.596570

[11] Soucy Chartier I, Provencher MD. Behavioural activation for depression: efficacy, effectiveness and dissemination. J Affect Disord. 2013;145(3):292–9. doi: 10.1016/j.jad.2012.07.023 22884236 10.1016/j.jad.2012.07.023

[12] Mazzucchelli TG, Kane RT, Rees CS. Behavioral activation interventions for well-being: a meta-analysis. J Posit Psychol. 2010;5(2):105–21. doi: 10.1080/17439760903569154 20539837 10.1080/17439760903569154 PMC2882847

[13] Mazzucchelli TG, Rees CS, Kane RT. Group behavioural activation and mindfulness therapy for the well-being of non-clinical adults: a preliminary open trial. The Cognitive Behaviour Therapist. 2009;2(4):256–71. doi: 10.1017/S1754470X09990201

[14] Berlim MT, Fleck MPA. Quality of life and major depression: current findings and future perspectives In: Ritsner MS, Awad AG, editors. Quality of life impairment in schizophrenia, mood and anxiety disorders. Netherlands: Springer; 2007 pp. 241–52. doi: 10.1007/978-1-4020-5779-3

[15] Yamamoto T, Shudo Y, Sakai M. Development of the Japanese version of behavioral activation for depression scale-short form (BADS-SF) and examination of its reliability and validity. Japanese Journal of Cognitive Therapy. 2015;8: 96–105.

[16] Radloff LS. The CES-D Scale: A self-report depression scale for research in the general population. Appl Psychol Meas. 1977;1:385–401. doi: 10.1177/014662167700100306

[17] Shima S, Shikano T, Kitamura T, Asai M. A new self-rating depression scale [in Japanese]. Clin Psychiatr. 1985;27:717–23.

[18] WHOQOL-Group. Development of the World Health Organization WHOQOL-BREF quality of life assessment. Psychol Med. 1998;28(3): 551–8. 9626712 10.1017/s0033291798006667

[19] Tazaki M, Nakane Y. WHOQOL-26 information Tokyo: Kaneko Shobou; 1997.

[20] Preacher KJ, Hayes AF. Asymptotic and resampling strategies for assessing and comparing indirect effects in multiple mediator models. Behav Res Methods. 2008;40:879–91. doi: 10.3758/BRM.40.3.879 18697684 10.3758/brm.40.3.879

[21] Baron RM, Kenny DA. The moderator-mediator variable distinction in social the moderator-mediator variable distinction in social psychological research: conceptual, strategic, and statistical considerations. J Pers Soc Psychol. 1986;51:1173–82. doi: 10.1037/0022-3514.51.6.1173 3806354 10.1037//0022-3514.51.6.1173

[22] Hopko DR, Lejuez CW, Ruggiero KJ, Eifert GH. Contemporary behavioral activation treatments for depression: procedures, principles, and progress. Clin Psychol Rev. 2003;23(5):699–717. doi: 10.1016/S0272-7358(03)00070-9 12971906 10.1016/s0272-7358(03)00070-9

[23] Mazzucchelli T, Kane R, Rees C. Behavioral activation treatments for depression in adults: a meta-analysis and review. Psychol Sci. 2009;16(4):383–411. doi: 10.1111/j.1468-2850.2009.01178.x

